# Hippocampal *TET1* and *TET2* Expression and DNA Hydroxymethylation Are Affected by Physical Exercise in Aged Mice

**DOI:** 10.3389/fcell.2018.00045

**Published:** 2018-04-19

**Authors:** Peter Jessop, Maria Toledo-Rodriguez

**Affiliations:** School of Life Sciences, University of Nottingham, Nottingham, United Kingdom

**Keywords:** TET1, TET2, 5hmC, exercise, aging, hippocampus

## Abstract

The function of 5-hydroxymethylcytosine (5hmC) is poorly understood. 5hmC is an epigenetic modification of DNA, resulting from the oxidation of 5-methylcytosine (5mC) by the Fe2^+^, and 2-oxoglutarate-dependent, 10–11 translocation methylcytosine dioxygenases (TET1, TET2, and TET3). Recent evidence suggests that, in addition to being an intermediate in active demethylation, 5hmC may also have an epigenetic role. 5hmC is enriched in the adult brain, where it has been implicated in regulating neurogenesis. The rate of adult neurogenesis decreases with age, however physical exercise has been shown to counteract this deficit. Here, we investigated the impact of voluntary exercise on the age-related changes of *TET1, TET2*, expression and 5hmC content in the hippocampus and hypothalamus. For this purpose, we used voluntary exercise in young adult (3 months) and aged (18 months) mice as a rodent model of healthy brain aging. We measured the levels of hippocampal and hypothalamic *TET1, TET2* mRNA, and 5hmC and memory [Object Location (OL) test] in mice that either exercised for 1 month or remained sedentary. While aging was associated with decreased *TET1* and *TET2* expression, voluntary exercise counteracted the decline in expression. Moreover, aged mice that exercised had higher hippocampal 5hmC content in the promoter region of *miR-137*, an miRNA involved in adult neurogenesis. Exercise improved memory in aged mice, and there was a positive correlation between 5hmC miR-137 levels and performance in the OL test. In the hypothalamus neither exercise nor aging affected *TET1* or *TET2* expression. These results suggest that exercise partially restores the age-related decrease in hippocampal *TET1* and *TET2* expression, which may be linked to the improvement in memory. Future studies should further determine the specific genes where changes in 5hmC levels may mediate the exercise-induced improvements in memory and neurogenesis in aged animals.

## Introduction

5′-methylcytosine (5mC) is a widely known and functionally well-understood epigenetic DNA modification. Less well-understood are the epigenetic modifications resulting from the iterative oxidation of 5mC by the Fe^2+^ and 2-oxoglutarate-dependent dioxygenases: translocation methylcytosine dioxygenases (TET1, TET2, and TET3) (Kriaucionis and Heintz, 2009; Tahiliani et al., 2009). These oxidation products are, sequentially: 5-hydroxymethylcytosine (5hmC) (Tahiliani et al., 2009), 5-formylcytosine (5fC), and 5-carboxylcytosine (5caC) (Ito et al., 2011). The TET enzymes are thought to be implicated in the active demethylation of DNA, as the enzyme TDG (Thymine-DNA glycosylase) can remove 5fC and 5caC from the genome via base excision (He et al., 2011). However, evidence suggests that, in addition to being an intermediate in active demethylation, 5hmC may also have an epigenetic role (Kriaucionis and Heintz, 2009). Interestingly, the highest levels of 5hmC within adult vertebrates are found in the brain, specifically in neurons (Globisch et al., 2010; Ruzov et al., 2011). The reason for this enrichment is currently unknown, but it may be a consequence of the neurogenic process (Zhang et al., 2013). When comparing neuronal precursor cells (NPCs) and mature neurons, there is a clear increase to 5hmC levels during differentiation (Zhang et al., 2013). Indeed, the accumulation of 5hmC in neuronal genes is positively correlated with increases in gene expression (Lin et al., 2017).

Differences to the genomic locations of 5hmC enrichment indicate that 5hmC plays multiple roles, varying by tissue type (Globisch et al., 2010) and developmental stage (Ruzov et al., 2011). This is unlike 5mC, which predominantly acts as a transcriptional repressor while, when located in the gene-body, 5mC can act as a transcriptional activator (Aran et al., 2011; Jones, 2012). For example, there is a stark difference in the genome-wide pattern of 5hmC between stem cells and brain cells, which might have a functional impact (Ficz et al., 2011; Pastor et al., 2011; Williams et al., 2011; Xu et al., 2011). In mouse embryonic stem cells, 5hmC is abundant at enhancers (Pastor et al., 2011; Wu et al., 2011; Yu et al., 2012) and at the transcriptional start sites of genes (TSS) (Ficz et al., 2011; Pastor et al., 2011; Williams et al., 2011; Xu et al., 2011). In neurons and brain tissues, 5hmC tends to be enriched within gene bodies (Xu et al., 2011). Interestingly a significant fraction of neuronal 5mC and 5hmC is located at non-CpG sites (where 5mC is predominantly deposited), namely at CpA dinucleotides (Jang et al., 2017). Additionally, 5hmC appears to play different roles according to the identity of the nucleotide flanking the modified cytosine (Kinde et al., 2015). This suggests that the potential roles of 5hmC in neurons may be different to other cell types.

In agreement with the high 5hmC levels in the brain, TET enzyme expression is also enriched (Hahn et al., 2013) and, together with 5hmC, have been shown to play a key role in neuronal differentiation and function (Santiago et al., 2014). For example, Zhang et al. recently showed that mice deficient in TET1, while still viable and fertile, showed decreased adult neurogenesis and impaired spatial learning and short-term memory (Zhang et al., 2013). Interestingly, while this study showed that *TET1* is highly expressed in neuronal progenitor cells, the negative effect of TET1 deficiency on neurogenesis was evident only in adulthood. These findings suggest that TET1 and 5hmC might be implicated in maintaining healthy levels of neurogenesis throughout the lifespan. A portion of the 5mC oxidized by TET enzymes is located within the genes of micro RNAs crucial for adult neurogenesis, such as RNA *miR-137*. Amongst others, *miR-137* regulates the levels of Ezh2, the histone methylase responsible for tri-methylating lysine 27 in histone 3 (H3K27me3), which defines the “poised state” (H3K27me3, H3K4me3) found in many neurogenic genes during neurogenesis (silent genes poised to be activated upon H2K27me3 demethylation).

The aging process is regulated by genetic and environmental factors (Govindaraju et al., 2015). While the genome determines the average life expectancy of a species, environmental factors, via epigenetic changes, regulate differences in longevity between individual members of a species [which can reach up to a 10-fold difference in honeybees and ants, (Kucharski et al., 2008)]. Aging is characterized by an alteration of epigenetic patterns or “epigenetic drift,” particularly affecting 5mC (Sierra et al., 2015). During aging there is a parallel genome-wide hypomethylation and promoter-specific hypermethylation (Jung and Pfeifer, 2015). Similarly, recent reports have indicated that there is a global increase of 5hmC in aged mice (Chen et al., 2012). However, the causal relationship between changes in epigenetic features in the brain, such as 5hmC, and the processes controlling aging are still not clear.

Physical exercise is recommended by the World Health Organization to improve and maintain health throughout the lifespan (World Health Organization, 2010). In the context of brain aging, it is known that exercise counteracts the decline in cognition and neurogenesis in old age (van Praag et al., 2005). Multiple studies have looked at the molecular mechanisms underlying the effects of exercise on brain health (Elsner et al., 2013; Morse et al., 2015). However, to our knowledge, there is no study focusing on the impact of voluntary exercise during aging on brain *TET1, TET2* expression, and 5hmC levels.

Here, we have studied the impact of voluntary exercise on the age-related changes to *TET1, TET2* expression, and 5hmC content in two different brain regions; the hippocampus and the hypothalamus. For this purpose, young adult and aged mice were allowed voluntary exercise for 1 month. Afterwards their memory was tested and their hippocampal and hypothalamic *TET1, TET2* mRNA and 5hmC levels measured. We found that voluntary exercise counteracts the age-related decrease in *TET1* and *TET2* expression, improves memory and increases *miR-137* 5hmC levels in the hippocampus of aged mice.

## Methods

### Animals, exercise, and object location test

Twenty-nine, C57BL/J6 male mice (fifteen 3 months old and fourteen 18 months old, purchased from Charles River, UK) were allowed to acclimatize to the animal facility for 1 week after arrival. Animals were then single housed and randomly distributed into two groups: exercise (young *n* = 8; aged *n* = 8) and sedentary (young *n* = 7; aged *n* = 6). Mice were on a 12 h light-dark cycle with food and water available *ad libitum*. Mice in the exercise group were housed with a running wheel for the entire duration of the study. The distances and time run by each animal were recorded daily. Mice in the sedentary group were housed with a non-functional wheel to control for environmental enrichment. After 4 weeks aged mice underwent the Object Location (OL) test, recognition of a familiar object moved to a novel location. This is a very mild behavioral test, which should not produce any stress or long-term changes of gene expression in the brain. Mice were habituated to a novel rectangular arena for 10 min. Afterwards mice were returned to their home cage for 5 min while the arena was cleaned and two identical objects were set in opposite corners of the same side of the arena. Then mice were returned to the arena and allowed to explore the objects for 6 min (habituation). After the habituation period, the mice were returned to their home cage for 5 min while the arena and objects were cleaned. One of the objects was placed in the original location while the other was moved to the across the arena (objects in opposite sides of the arena). Animals were returned to the arena and allowed to freely explore the objects for 6 min (OL). Object investigation was manually recorded using Clicker software (v1.13). The investigation ratio was calculated as the percentage of the total exploration time spent investigating the object in the novel location vs. time exploring both objects. Five weeks after the start of the experiment, mice were humanely killed by cervical dislocation, their brains extracted and then the hippocampus and hypothalamus were dissected and immediately frozen. This study was carried out in accordance with the license authorities issued under the UK Animals (Scientific procedures) Act 1986. The project was approved by the University of Nottingham Animal Welfare and Ethical Review Body.

### Gene expression profiling by Q-PCR

RNA was isolated with the AllPrep DNA/RNA/Protein mini Kit (Qiagen, Germany) following the manufacturer's instructions. 500 ng of RNA were reverse transcribed with Superscript III (Life Technologies, UK) and 15-mer random primers (Sigma, UK). Q-PCR reactions were then performed in triplicates with the SensiMix Plus SYBR Green PCR kit (Bioline, UK) in a RotorGene 6000 cycler (Qiagen, Germany). PCR primers for *TET1* [NM_001253857.2] [forward primer 5′-ATC ATT CCA GAC CGC AAG AC-3′] and reverse primer 5′-AAT CCA TGC AAC AGG TGA CA-3′] and *TET2* [NM_001346736.1] [forward primer 5′-CCA CAG AGA CCA GCA GAA CA-3′ and reverse primer 5′-TCC GCT TTC TTC TTG CAA CT-3′] were designed with the Primer3 software (http://primer3.ut.ee/). Primer's efficiency and specificity was verified using melting curve analysis (single melt curve peak). For the hippocampus, gene expression was normalized to the geometric average of the control genes [*Pgk1, TATA-box binding protein (TBP)* and *Hprt1*] according to the GeNorm normalization (Vandesompele et al., 2002), for the hypothalamus, gene expression was normalized to the control gene *Pgk1* (as other housekeeping gene candidates were affected by the treatment). Primers for *Pgk1* [NM_008828.3] [forward primer 5′-GAA GGG AAG GGA AAA GAT GC-3′ and reverse primer 5′-AAA TCC ACC AGC CTT CTG TG-3′], *TBP* [NM_013684.3] [forward primer 5′-CAG CCT TCC ACC TTA TGC TC-3′ and reverse primer 5′-TGC TGC TGT CTT TGT TGC TC-3′], and *Hprt1* [NM_013556.2] [forward primer 5′-GCA GTA CAG CCC CAA AAT GG-3′ and reverse primer 5′-AAC AAA GTC TGG CCT GTA TCC A-3′]. The relative expression levels of each mRNA were calculated using a modified 2 Delta-Delta-Ct algorithm (Pfaffl, 2001).

### MeDIP

Genomic DNA was isolated with the AllPrep DNA/RNA/Protein mini Kit (Qiagen, Germany) following the manufacturer's instructions. Quality and quantity of the gDNA was assessed with nanodrop (ThermoFisher, UK). For 5hmC analysis, 1 ug per sample was sonicated using a Bioruptor (Diagenode, Belgium). Two hundred nanograms from each sample were then removed to serve as an input control before MeDIP was performed. The remaining sonicated gDNA was precipitated using rabbit-anti-5hmC antibody (39769, Active Motif, CA, USA) followed by incubation with anti-rabbit Dynabeads (ThermoFisher Scientific, UK). Afterwards, the samples were digested with proteinase K (P2308, Sigma, UK) followed by phenol-chloroform extraction and ethanol precipitation. Q-PCR reactions were then performed in triplicates with the SensiMix Plus SYBR Green PCR kit (Bioline, UK) in a RotorGene 6000 cycler (Qiagen, Germany). PCR primers for mature *miR-137-3p* [NM_008173.3] [forward primer 5′-GAT TTA TGG TCC CGG TCA AG-3′ and reverse primer 5′-AAT ACC CGT CAC CGA AGA GA-3′] were designed with the Primer3 software (http://primer3.ut.ee/). The *miR-137* sequence was obtained from www.mirbase.org This sequence contains the Neuron-Restrictive Silencer Element NRSE/RE1 response element (Warburton et al., 2015), which has been shown to be associated with inactivation and, recently, activation of non-neuronal gene expression (Zhao et al., 2017) (see **Figures 2C,D** for site and sequence information, respectively). Primer efficiency and specificity were verified using melting curve analysis (single melt curve peak). Enrichment of methylated DNA was calculated using the method detailed by Taiwo et al. (2012). A parallel MeDiP study, using rabbit-anti-5mC antibody (61255, Active Motif, CA, USA), was performed to investigate the impact of exercise on hippocampal 5mC content.

### 5hmC ELISA

Total 5hmC DNA levels were measured using the Quest 5hmC DNA ELISA Kit (D5425, Zymo Research, USA) following the manufacturer's instructions. In brief, 50 ng from each sample were tested in duplicates or triplicates using a sandwich design. Anti-5hmC antibodies were bound to the ELISA plate, afterwards genomic DNA was incubated followed by anti-DNA HRP conjugated antibodies and developer. The 405–450 nm absorbance was measured on a TECAN “sunrise” basic ELISA model (TECAN, Austria).

### Statistics

Q-PCR and ELISA data were analyzed using two-way ANOVA (for single comparison between groups we used Student's *t*-test). MeDIP and OL results were analyzed using Student's *t*-test. In all cases, normality of the data distribution was confirmed by d'Agostino test. Pearson's correlation was employed to study the relationship between gene expression, 5hmC or 5mC levels and OL results. Analyses were performed with Prism (version 6 GraphPad Software, USA). Statistics reported in the text and figures represent the mean ± S.E.M. For all tests, null hypotheses were rejected at probability level of *p* < 0.05.

## Results

Using a rodent model of long-term voluntary exercise, we studied the impact of physical exercise on memory, 5mC, 5hmC levels, and *TET1* and *TET2* expression in the hippocampus and hypothalamus of young adult and aged mice.

### Aging decreased hippocampal *TET1* and *TET2* expression, which was rescued by physical exercise

**Figure 1:**
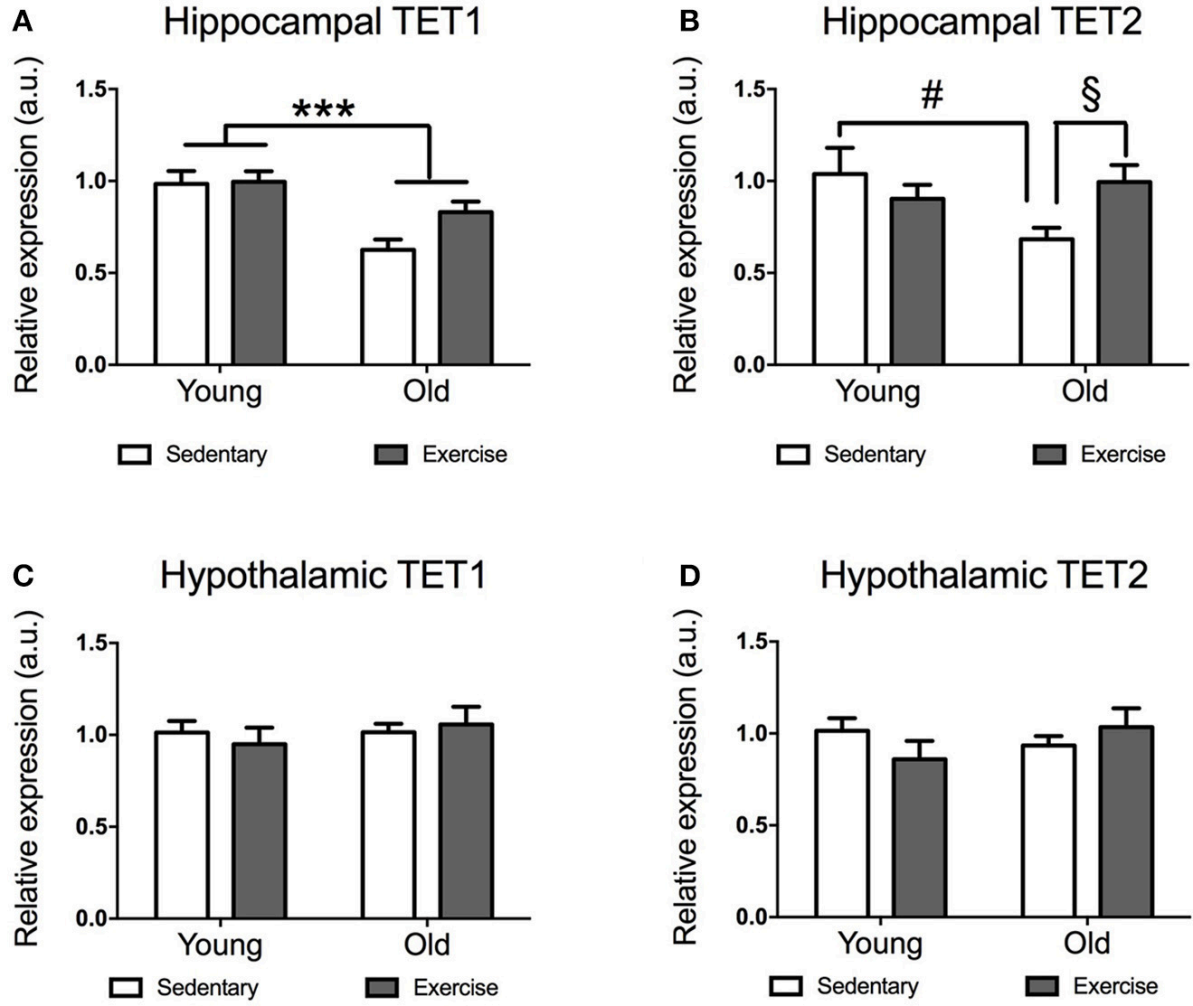
Impact of exercise and aging on *TET1, TET2* expression in the hippocampus and hypothalamus. *TET1* **(A,C)** and *TET2* **(B,D)** expression in hippocampus **(A,B)** and hypothalamus **(C,D)** for young and aged mice that exercised or remained sedentary. Young sedentary *n* = 5–6, young exercise *n* = 7–8, old sedentary *n* = 5–6, and old exercise *n* = 7–8. ^***^p < 0.001; ^#^*p* < 0.05 *post-hoc* comparison between sedentary aged mice and sedentary young mice; ^§^*p* < 0.05 *post-hoc* comparison between aged mice that exercise and aged mice that remained sedentary.

Both, young and aged mice spontaneously run after getting access to the wheels, increasing their daily running distance until ~10 days (when they run similar distances day after day). While young mice reached average running distances of 5–15 km a day, aged mice run distances of 0.5–4 km a day (data not shown). Aged mice expressed significantly lower levels of *TET1* mRNA in their hippocampi [*F*_(1, 21)_ = 17.86 *p* = 0.0004] (Figure 1A). The decrease in *TET1* expression was more pronounced in the sedentary aged mice, while it seems that exercise leads to a recovery of *TET1* levels in the aged mice. There was a significant age^*^exercise interaction in *TET2* expression [*F*_(1, 22)_ = 5.281 *p* = 0.0314]. *Post-hoc* comparisons indicated that sedentary aged mice had significantly lower *TET2* expression than sedentary young mice [*t*_(10)_ = 2.28 *p* = 0.0458] (Figure 1B) and exercise-aged mice [*t*_(11)_ = 2.673 *p* = 0.0217] (Figure 1D).

### Neither aging nor exercise affected *TET1* or *TET2* expression levels in the hypothalamus

**Figure 2:**
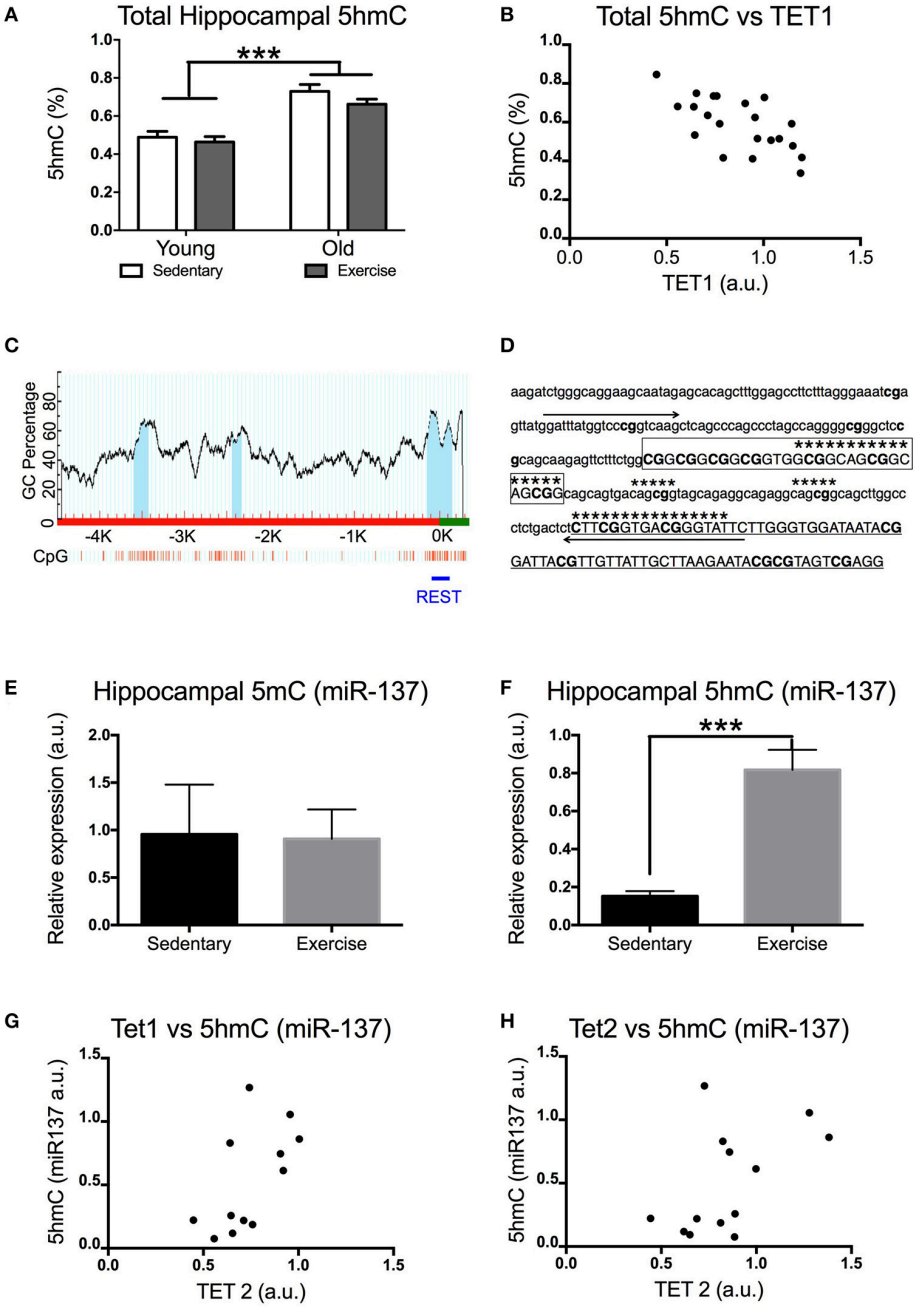
Impact of exercise and aging on *total 5hmC, 5mC(miR-137), and 5hmC (miR-137)* levels in the hippocampus. **(A)** Total hippocampal 5hmC levels. **(B)** Correlation between total 5hmC and TET1 expression in the hippocampus. **(C)** Scheme of the *miR-137* promoter region CpG islands (dark area under the curve) in promoters and 5′-untranslated regions of *miR-137* (obtained with http://www.urogene.org/methprimer, individual CpGs are depicted by vertical lines). A horizontal line indicates the region whose DNA methylation was studied by MeDIP. **(D)** Sequence targeted by PCR primers for the internal *miR-137* promoter. **(E)** 5mC and **(F)** 5hmC enrichment for *miR-137* (NRSE/RE1 region) in aged mice that exercised or remained sedentary. Correlation between 5hmC (miR-137) levels and hippocampal expression of **(G)** *TET1* or **(H)** *TET2* in aged mice. Young sedentary *n* = 5–6, young exercise *n* = 7–8, old sedentary *n* = 5–6, and old exercise *n* = 7–8. ^***^p < 0.001.

Unlike in the hippocampus, we did not find any significant effect of age or exercise on *TET1* or *TET2* expression in the hypothalamus (Figures 1C,D). However, there was a tendency for decreased expression of *TET2* in aged mice, which seems to be restored by exercise (Figure 2B).

### Global hippocampal 5hmC levels increase with age and are negatively correlated with TET1 expression

An increase in *TET1* and *TET2* expression might result in increased levels of 5hmC (if 5mC is oxidized to 5hmC) or a decrease in 5hmC levels (if TET enzymes further oxidize 5hmC into 5fC and/or 5caC). Using ELISA we measured the total hippocampal 5hmC content. Aged mice had significantly higher levels of 5hmC in their hippocampi [*F*_(1, 21)_ = 50 *p* = 0.0001] (Figure 2A). Interestingly, there was a significant negative correlation between the levels of 5hmC and TET1 expression in the hippocampus. [Pearson's *r*_(21)_ = −0.661, *p* = 0.0010] (Figure 2B).

### Physical exercise significantly increased hippocampal 5hmC content on *miR-137* internal promoter

It is known that the impact of aging on 5mC levels differs depending on the gene. In order to find out whether this applies to 5hmC we studied the effect of exercise on 5mC and 5hmC levels in the internal promoter region of *miR-137* in the hippocampus of aged mice (Figure 2B). This region contains the NRSE/RE1 response element (Figures 2C,D). *miR-137* was chosen for this study due to its crucial role in a feedback loop regulating adult neurogenesis (which also includes MeCP2 and Ezh2). Previous research has shown that adult neurogenesis decreases with aging and voluntary exercise counteracts this effect. While voluntary exercise had no effect on the *miR-137* 5mC levels of aged mice [*t*_(10)_ = 0.08454 *p* = 0.9343] (Figure 2E), it resulted in a significant increase in the amount of hippocampal *miR-137* 5hmC [*t*_(12)_ = 5.289 *p* = 0.0002] (Figure 2F). Additionally, we found significant positive correlation between the levels of 5hmC in miR-137 and *TET1* expression in the hippocampus [Pearson's *r*_(12)_ = 0.598, *p* = 0.0400] (Figure 2G). The correlation between the levels of 5hmC in *miR-137* and *TET2* expression in the hippocampus was not significant [Pearson's *r*_(13)_ = 0.5244, *p* = 0.0658] (Figure 2H).

### Physical exercise significantly increased memory (object location) in aged mice

**Figure 3:**
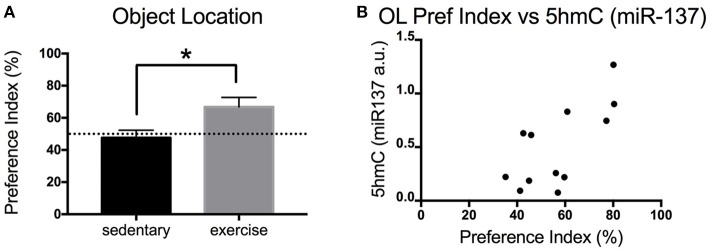
Impact of exercise on memory (OL) in aged mice. **(A)** Preference index for the OL test for young and aged mice that exercised or remained sedentary. **(B)** Correlation between hippocampal 5hmC (*miR-137*) levels and preference index in the OL test. Old sedentary *n* = 5 and old exercise *n* = 6. ^*^p < 0.05.

Exercise is known to increase adult neurogenesis in aged animals. Increased adult neurogenesis is related to improved memory. Here, we measured the impact of physical exercise on the memory of aged mice using the OL test. Mice that exercise showed a significant increase in the preference index (exploration) of the novel object [*t*_(9)_ = 2.457 *p* = 0.0363] (Figure 3A). Interestingly, there was a significant positive correlation between the preference index of the mice and their levels of hippocampal *miR-137* 5hmC [Pearson's *r*_(12)_ = 0.6965, *p* = 0.0119] (Figure 3B).

## Discussion

By 2050, worldwide, 1 in 4 people will be 65 years or older (EBioMedicine, 2015). However, this increase in lifespan is not translating into an increase in healthspan (disease free years) (EBioMedicine, 2015). Indeed, with increasing age, the maintenance of good health becomes progressively more challenging, affecting physical as well as mental health. For example, age is the biggest risk factor for the development of neurodegenerative disorders (Niccoli and Partridge, 2012). Maintaining an active lifestyle with regular exercise is known to be beneficial for mental health and, when combined with cognitive training, helps improve neurocognitive function in older adults (Smith et al., 2010; Law et al., 2014). Multiple studies have looked at the molecular mechanisms underlying the effects of exercise on healthy brain aging (Elsner et al., 2013; Morse et al., 2015). However, to our knowledge, no study has focused on the impact of voluntary exercise on expression levels of *TET1, TET2*, and 5hmC in young and aged brains.

Here, we have investigated the effects of physical exercise on hippocampal *TET1* and *TET2* expression during aging. For this purpose, we used: (a) voluntary exercise in young adult (3 months) and aged (18 months) mice as a rodent model of healthy brain aging and (b) Q-PCR to measure gene expression. We compared the age-related changes in animals that exercised or remained sedentary. We showed that exercise in mice caused a halt to the age-related decline in *TET1* and *TET2* expression in the hippocampus, while there were no significant differences in the hypothalamus.

Two previous studies of *TET1* and *TET2* expression levels in mouse brain tissues suggested a lack of significant changes during postnatal neurodevelopment (Chen et al., 2012; Hadad et al., 2016) however, this data was obtained comparing mice older than our study (24 months old instead of 18 months old). While the age bracket for “old” or “aged” C57BL/6J mice is 18–24 months old, at 24 months there is sharp increase in strain-specific diseases, which can lead to changes in gene expression and thus impact the study outcomes (Flurkey et al., 2007; Moeller et al., 2014). Moreover, both studies use a single housekeeping gene to normalize the Q-PCR results. This can be a confounding factor as, in some, instances the levels of housekeeping gene expression can vary considerably (Suzuki et al., 2000). In our study, we have avoided this by using a combination of three housekeeping genes and GeNorm analysis to compute the normalization factor values, which confirmed that neither age nor exercise affected them (Vandesompele et al., 2002). This strategy enables reliable normalization of Q-PCR data by identifying and employing the most stable control genes to generate a robust normalization factor.

One functional implication of the exercise-induced normalization of hippocampal *TET1* and *TE2* expression could be changes in adult neurogenesis. Previous studies have shown that TET1 and TET2 are involved in the regulation of neurogenesis (Hahn et al., 2013; Zhang et al., 2013; Santiago et al., 2014) and neuronal activity (Szulwach et al., 2011; Rudenko et al., 2013; Perera et al., 2015). For example, while *TET1* knock down does not impact the development or reproduction of mice, it reduces the amount of neuronal progenitor cells in the adult hippocampus and impairs learning and memory (Zhang et al., 2013). Additionally, *TET2* knock down results in defective differentiation of newly generated neurons (Hahn et al., 2013). Thus, we could argue the aged-induced downregulation in *TET1 and TET2* expression could be implicated in the decrease to neurogenesis in old age. A restoration of *TET1* and *TET2* expression levels could result in a recovery of adult neurogenesis. This could provide a mechanistic explanation to the exercise-induced restoration of adult neurogenesis in aged mice (van Praag et al., 2005). Indeed, in this study we showed that aged mice that exercised had improved memory (measured by the OL test) and that the memory (preference index) negatively correlates with the levels of hippocampal 5hmC.

*TET1* and *TET2* sequentially oxidize 5mC into 5hmC, 5fC, and 5caC (Tahiliani et al., 2009; Ito et al., 2011). Thus we studied whether the impact of aging and physical exercise on *TET1* and *TET2* resulted in changes in hippocampal 5hmC levels. Interestingly we found that aging resulted in a significant increase of hippocampal 5hmC. Indeed, the significant negative correlation between total 5hmC levels and *TET1* expression seems to suggest that the lower levels of TET1 in old animals may result in decreased demethylation. However further studies should investigate whether this is the case. While at first glance, an increase in TET1 expression is expected to result in an increase of 5hmC levels, that may not be the case if TET1 further oxidizes 5hmC into 5fC and 5caC.

Similar to the impact of aging on 5mC levels we also found that the impact of aging and exercise on levels of 5hmC differ depending on the gene studied. The exercise-related changes to *TET1* and *TET2* expression in aged hippocampi of old mice were accompanied by an approximately 3-fold increase to the level of 5hmC at the internal promoter of the neurogenic factor, *miR-137*. Additionally, the correlation analyses we performed suggest that the degree of hydroxymethylation at the *miR-137* promoter region interrogated is higher in hippocampi with higher *TET1* and *TET2* expressions. Irier et al. recently described that environmental enrichment reduces 5hmC content in the hippocampus of aged mice (Irier et al., 2014). However, while their environmental enrichment protocol contained a running wheel, the mice only had access to the wheels for 3 h a day during the light phase of the light/dark cycle. Mice and other nocturnal animals rarely run during the inactive light phase (0.0–0.1 km per day vs. 5–15 km per night for young mice, data not shown) (Goh and Ladiges, 2015). Thus, it is unlikely that the mice in Irier's study did much running (Irier et al., 2014). In our study, mice were housed with the running wheel for a month and thus could run voluntarily during the entire dark phase of the cycle. Moreover, in Irier's study there was only a single running wheel for the 4 mice in the group (Irier et al., 2014). This may have caused fights between the mice and the resulting stress may have confounded the results. In our experiment, each mouse was housed with its own running wheel.

The exercise-induced increase in 5hmC during aging could mediate, in part, the recovery in adult neurogenesis levels previously reported in aged mice (van Praag et al., 2005). Indeed, in embryonic stem cells, neuronal differentiation is correlated with increase in 5hmC levels, especially at gene bodies of neuron-specific genes (Hahn et al., 2013). In our study we found that exercise increased 5hmC content at the internal promoter region of *miR-137*, a neurogenic miRNA, and this increase is correlated with an improvement in memory. *miR-137* is an important regulator of cellular proliferation and differentiation, through regulating translation of the histone methylase, *EZH2*, thus influencing H3K27 tri-methylation (Mahmoudi and Cairns, 2017). Therefore, change in hydroxymethylation at the *miR-137* internal promoter could affect neurogenesis levels by modifying *miR-137* expression. Additionally, an increase in *miR-137* promoter hydroxymethylation could be implicated in regulating aged-related neuroinflammation. In agreement with this, a recent study has shown that *miR-137* overexpression inhibits the inflammatory reaction resulting from spinal cord injury (Gao et al., 2017). The *miR-137* region we studied contains the NRSE/RE1 response element (Warburton et al., 2015). The impact of increased 5hmC levels at the *miR-137* NRSE/RE1 region is more difficult to interpret due to the complexity of its ligand's actions (RE-1 Silencing Transcription factor, REST/NRSF). While it is widely known that REST/NRSF regulates the expression of non-neuronal genes through binding to the silencer element NRSE/RE1, recent studies have shown that REST/NRSF is also an activator inducing neuronal differentiation (Zhao et al., 2017). Thus, further studies should explore the functional implications of the increase to hydroxymethylation at the NRSE/RE1 in *miR-137*.

Understanding whether there is a causative link between the exercise-induced increases to *TET1* and *TET2* expression, 5hmC levels and neurogenesis will require further studies. A future experiment could employ the same rodent model of voluntary exercise using homozygous knockout mice for *TET1* and/or *TET2*. Changes in neurogenesis, *miR-137* expression and hydroxymethylation patterns at the *miR-137* gene could then be examined. As our experiment used the whole hippocampus, future studies in old animals should determine the location and cell type(s) within the hippocampus where *TET1, TET2* expression, and 5hmC level changes take place in response to exercise. Moreover future studies should investigate the differential impact of exercise and aging on 5hmC in different genes.

## Author contributions

PJ: participated in the design and coordination, carried out the gene expression and MeDIP studies, performed statistical analysis and helped to draft the manuscript; MT-R: conceived the study, participated in the design and coordination, carried out the animal and ELISA studies, performed statistical analysis and drafted the manuscript. All authors read and approved the final manuscript.

### Conflict of interest statement

The authors declare that the research was conducted in the absence of any commercial or financial relationships that could be construed as a potential conflict of interest. The handling Editor declared a shared affiliation, though no other collaboration, with the authors PJ, MT-R.
